# Differences in catalase levels between malaria-infected individuals and uninfected controls: a systematic review and meta-analysis

**DOI:** 10.1038/s41598-023-41659-4

**Published:** 2023-09-05

**Authors:** Manas Kotepui, Aongart Mahittikorn, Frederick Ramirez Masangkay, Kwuntida Uthaisar Kotepui

**Affiliations:** 1 Medical Technology, School of Allied Health Sciences, Walailak University, Tha Sala, Nakhon Si Thammarat, Thailand; 2 Department of Protozoology, Faculty of Tropical Medicine, Mahidol University, Bangkok, Thailand; 3 Department of Medical Technology, Faculty of Pharmacy, University of Santo Tomas, Manila, Philippines

**Keywords:** Diagnostic markers, Infectious diseases, Malaria

## Abstract

Inconsistent catalase (CAT) research necessitates a comprehensive review of CAT levels among patients with malaria to achieve better therapeutic strategies. This study aimed to systematically review and meta-analyze available literature on CAT levels in nonpregnant and pregnant individuals with malaria compared with those in uninfected controls, with the goal of providing a robust evidence base for future research and potential interventions. Following PRISMA guidelines, a systematic literature search across six databases was conducted to examine CAT levels in patients with malaria. Data was extracted independently by two reviewers, and study quality was assessed using the Joanna Briggs Institute (JBI) critical appraisal checklist. The standardized mean difference of CAT levels was calculated with heterogeneity assessment. Subgroup and sensitivity analyses were conducted to explore heterogeneity and assess the robustness of the findings. Publication bias was visually and statistically assessed and corrected, if necessary. Statistical analyses were performed using Stata software, with a significance level set at *P* < 0.05. Nineteen studies were included in the review. These studies, published from before 2000 to 2023, primarily from Africa and Asia, focused on different *Plasmodium* species and age groups. Results of qualitative synthesis among nonpregnant individuals consistently showed lower CAT levels in malaria-infected individuals, although some studies reported higher levels. No significant differences in CAT levels were found between malaria-infected and uninfected individuals, as demonstrated by a meta-analysis overall (*P* = 0.05, Hedges’ g: − 0.78, 95% confidence interval (CI): (− 1.56)–0.01, I^2^: 98.47, 15 studies), but subgroup analyses showed significant differences in CAT levels in studies conducted in Africa (*P* = 0.02, Hedges’ g: − 0.57, 95% CI: − 1.02–(0.11), I^2^: 91.81, 7 studies), and in studies that specifically focused on children (*P* = 0.03, Hedges’ g: − 0.57, 95% CI: − 1.07–(− 0.07), I^2^: 87.52, 4 studies). Pregnant women showed variations in CAT levels across trimesters. This study provides valuable insights into the association between malaria infection and CAT enzyme levels, particularly in nonpregnant individuals. Furthermore, well-designed studies are essential to decoding the intricacies of this relationship, which could have significant implications for understanding disease processes and improving patient care.

## Introduction

Malaria, a life-threatening disease caused by *Plasmodium* parasites, continues to be a significant global health concern^1^. The disease primarily impacts vulnerable populations, particularly in sub-Saharan Africa and Southeast Asia^2–5^. Of particular concern is the burden of malaria among women, with pregnant women and their unborn children facing considerable risks, including maternal anemia, low birth weight, and increased infant and maternal mortality^6–8^. The human body’s response to malaria infection is an intricate and multifaceted process involving the immune system and oxidative stress^9,10^. The immune response plays a crucial role in controlling the initial stages of infection and reducing parasitemia levels^11^. Upon infection, the immune system triggers the release of proinflammatory cytokines, such as tumor necrosis factor-alpha (TNF-α), interleukin-1 (IL-1), and interferon-gamma (IFN-γ), which are intended to suppress parasite growth and proliferation^12^. However, this defensive response may inadvertently exacerbate disease severity due to the associated inflammation and tissue damage^13^.

Oxidative stress, which results from an imbalance between the production of reactive oxygen species (ROS) and the body’s antioxidant defense, has emerged as a key player in the pathophysiology of malaria^14–16^. ROS, including superoxide anions, hydroxyl radicals, and hydrogen peroxide, are produced during normal metabolic processes but can lead to cellular damage when their levels surpass the body’s antioxidant capacity. In malaria, *Plasmodium* parasites have been shown to increase ROS production, leading to oxidative stress and contributing to the clinical manifestations of the disease^16^. The antioxidant defense system, which is the system that neutralizes ROS by catalyzing their conversion into less reactive substances, and includes enzymes, such as superoxide dismutase (SOD), glutathione peroxidase (GPx), and catalase (CAT), is the body’s primary line of defense against the harmful effects of ROS^17^. Furthermore, *Plasmodium* infection can deplete the glutathione levels in the host’s red blood cells^18^. As glutathione is a crucial intracellular antioxidant that helps maintain the redox balance in cells, the depleted glutathione levels can make red blood cells more susceptible to damage by ROS, which could further exacerbate the symptoms of malaria or lead to complications^9^.

CAT, a vital enzyme in the body’s antioxidant defense system, plays a pivotal role in neutralizing harmful ROS, particularly hydrogen peroxide^19^. Notably, several pathogens, including bacteria, fungi, and parasites may manifest altered CAT levels. These microorganisms can co-opt CAT to counteract oxidative stressors, thereby boosting their resilience and survival within the host^20,21^. For instance, some studies have documented elevated CAT blood levels in children diagnosed with bacterial meningitis^22^, whereas another study observed a decrease in CAT levels within a similar patient group^23^. The interplay between malaria and other infectious diseases, particularly about varying CAT levels, presents a fascinating area for exploration. One could speculate that malaria, by modulating CAT levels, could heighten the vulnerability of individuals, notably children, to secondary bacterial infections due to a weakened antioxidant defense mechanism. Thus, evaluating CAT levels in patients with malaria not only provides deeper insights into its impact on oxidative stress but also carries broader therapeutic implications for both malaria and associated conditions like bacterial infections.

However, the current landscape of research on CAT levels in patients with malaria reveals inconsistencies and gaps, particularly concerning specific demographics like pregnant and nonpregnant individuals and spanning different disease severities. This emphasizes the need to obtain a comprehensive overview of CAT dynamics in the context of malaria. Against this background, this study was implemented to systematically review and meta-analyze literature on CAT levels in both nonpregnant and pregnant individuals afflicted with malaria compared to uninfected controls, with the goal of establishing a broader perspective and stronger foundation to spur future research and therapeutic interventions.

## Methods

The protocol of systematic review was registered at PROSPERO (CRD42023430979). The reports of a systematic review and meta-analysis followed the Preferred Reporting Items for Systematic Reviews and Meta-Analyses (PRISMA) statements^24^.

### Search strategy

A comprehensive and systematic literature search was conducted across six databases, namely, Embase, MEDLINE, Ovid, PubMed, Scopus, and ProQuest, up to May 2023. The keywords used in this search were “malaria,” and “catalase,” and variations thereof (Table S1). Bibliographies of identified studies and Google Scholar were also manually searched to identify any potentially eligible studies not found in the database search.

### Selection criteria

Studies were included in this systematic review and meta-analysis if they met the following criteria: (i) the study examined humans infected with malaria (*P. falciparum*, *P. vivax*, or both), (ii) the study measured CAT levels in blood or plasma, (iii) the study provided data on CAT levels in patients with malaria and uninfected controls, and (iv) the report on the study was published in English. Exclusion criteria were as follows: (i) in vitro or animal studies, (ii) reviews, conference abstracts, or duplicate reports, (iii) studies without an uninfected group, and (iv) studies that did not provide data on CAT levels. Two authors (MK, AM) identify relevant studies independently, with disagreements resolved through discussion.

### Data extraction and quality assessment

Two independent reviewers (MK, AM) extracted data from eligible studies using a standard extraction form, with disagreements resolved through discussion. Extracted data included: first author’s name, year of publication, country (year of conduction), study design, age group of participants, *Plasmodium* species, number of participants (infected and controls), clinical status, mean or median CAT level, and standard deviation or interquartile range of CAT levels, diagnostic methods for malaria, and test for CAT.

The quality of the included studies was assessed using the Joanna Briggs Institute (JBI) critical appraisal checklist for cross-sectional, cohort, and case–control studies^25^. The cross-sectional studies checklist assesses clear inclusion criteria, subject and setting details, condition identification and measurement reliability, confounding factors, outcome measurement, and statistical analysis. The cohort studies checklist focuses on group similarity, exposure measurement, confounding factors, follow-up periods, outcome measurement, and statistical analysis. The case–control studies checklist ensures group comparability, matching appropriateness, identification criteria, exposure measurement, handling of confounding factors, and statistical analysis. Each question can be answered with “Yes,” “No,” “Unclear,” or “Not applicable,” providing a systematic framework to evaluate a study’s reliability and quality. The quality of studies was assessed by two authors independently (MK, KUK), with disagreements resolved through discussion.

### Statistical analysis

The standardized mean difference of CAT levels between patients with malaria and uninfected controls was calculated as Hedges’ g, with 95% confidence intervals (CIs). Heterogeneity was assessed using I^2^ statistic and considered substantial if I^2^ was > 50%^26^. The random-effects model was applied in the presence of substantial heterogeneity^27^. The meta-regression and subgroup analyses were conducted based on publication year, study design, continent, age group, *Plasmodium* species, clinical status, and method for diagnosing malaria to explore potential sources of heterogeneity.

To evaluate the robustness of the findings, a sensitivity analysis was performed by omitting one study at a time and examining the influence of each individual study on the pooled estimate^28^. The presence of publication bias was assessed visually using funnel plots^29^ and statistically through Egger’s test^30^. If asymmetry was detected, the trim-and-fill method was employed to correct for potential publication bias^31^. All statistical analyses were performed using Stata software version 17.0 (StataCorp, Texas, USA). All *P* values were two-tailed, and significance was set at < 0.05.

## Results

### Search results

**Figure 1:**
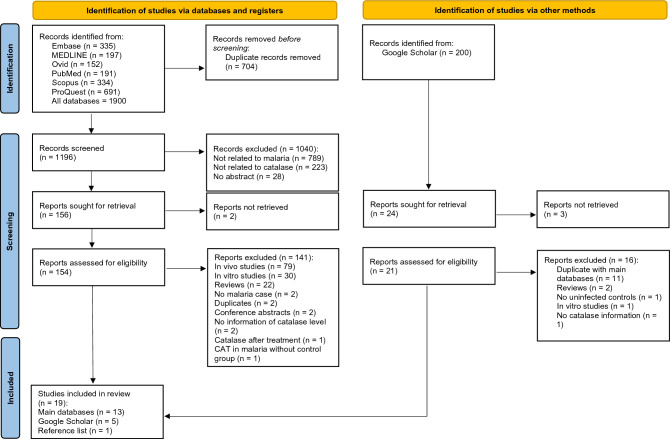
Study flow diagram.

The initial records were identified from various databases as follows: Embase (n = 335), MEDLINE (n = 197), Ovid (n = 152), PubMed (n = 191), Scopus (n = 334), and ProQuest (n = 691). The total number of records across all databases was 1900. Before screening, duplicate records were removed, resulting in the elimination of 704 duplicate records. After the removal of duplicates, the remaining records were screened. The total number of records screened was 1196. During the screening process, certain records were excluded for specific reasons. Out of the 1196 records screened, 1040 were excluded. The reasons for exclusion were as follows: 789 records were not related to malaria, 223 records were not related to catalase, and 28 records had no abstract. After the screening process, reports were sought for retrieval. Out of the initially screened records, 156 reports were sought for retrieval. During the retrieval process, 2 reports could not be retrieved. The retrieved reports were assessed for eligibility, resulting in 154 reports being assessed. Out of these 154 assessed reports, 141 were excluded for the following reasons: 79 reports were on in vivo studies, 30 reports were on in vitro studies, 22 reports were reviews, 2 reports did not mention any malaria cases, 2 reports were duplicates, 2 reports were conference abstracts, 2 reports had no information on catalase level, 1 report involved catalase after treatment, and 1 report examined CAT in malaria without a control group. In the end, 19 studies were included in the review^14,32–49^. Out of these, 13 studies were from the main databases^14,32–36,38,40,41,43,46–48^, 5 studies were from Google Scholar^37,39,42,45,49^, and 1 study was identified through a reference list^44^ (Fig. 1).

### Characteristics of studies

The dataset analyzed in this academic context consists of 19 studies (Table S2). The studies were published between before 2000 and 2023, with the majority (57.9%) being published from 2010 onwards. The study designs employed include cross-sectional studies (79.0%), cohort studies (10.5%), and case–control studies (10.5%). Most of the included studies was conducted in Africa (57.9%), predominantly in Nigeria, Cameroon, Ghana, and Uganda; and in Asia (31.6%), primarily in India and Turkey. Europe (France) and South America (Colombia) were also represented with one study each (5.26%). The studies focused on different species of *Plasmodium*, the parasite responsible for malaria. The majority of studies (73.7%) examined *P. falciparum*, whereas a smaller proportion investigated both *P. falciparum* and *P. vivax* (10.5%) or solely *P. vivax* (15.8%). The participants in these studies were categorized into different groups, with a focus on children (26.3%), adults (41.4%), or all age groups (15.8%). Some studies did not specify the age range of the participants (10.5%). Clinical status was also considered, with the prevalence of symptomatic malaria (73.7%), both symptomatic and asymptomatic malaria (10.5%), and studies that did not define the malaria status of participants (15.8%). The methods utilized for malaria detection included microscopy (84.2%) and a combination of microscopy and rapid diagnostic tests (RDTs) (15.8%) (Table 1).

**Table 1:** Characteristics of studies.

Characteristics	N (19 studies)	**%**
Publication year		
2010–2023	11	57.9
2000–2009	5	26.3
Before 2000	3	15.8
Study design		
Cross-sectional studies	15	79.0
Cohort study	2	10.5
Case–control studies	2	10.5
Study area		
Africa	11	57.9
Nigeria	6	
Cameroon	3	
Ghana	1	
Uganda	1	
Asia	6	31.6
India	4	
Turkey	2	
Europe (France)	1	5.26
South America (Colombia)	1	5.26
*Plasmodium* spp.		
* P. falciparum*	14	73.7
* P. falciparum, P. vivax*	2	10.5
* P. vivax*	3	15.8
Participants		
Children	5	26.3
Adults	9	41.4
All age groups	3	15.8
Not specified	2	10.5
Clinical status		
Symptomatic malaria	14	73.7
Symptomatic and asymptomatic malaria	2	10.5
Not defined	3	15.8
Methods for malaria detection		
Microscopy	16	84.2
Microscopy/RDT	3	15.8

*RDT* rapid diagnostic test.

### Risk of bias

Overall, the included studies exhibited varying levels of methodological rigor, with some demonstrating more robust methodologies (Table S3). Among the examined cross-sectional studies, three studies^32–34^ demonstrated notable methodological strengths, including well-defined inclusion criteria, comprehensive subject and setting descriptions, valid exposure measurements, and objective criteria for assessing the investigated condition. However, these studies lacked sufficient consideration of potential confounding factors and strategies to mitigate their impact. In contrast, three studies^38–40^ successfully identified confounding factors and implemented strategies to address them. All studies exhibited valid and reliable outcome measurements, with most employing appropriate statistical analysis. The particular statistical analysis applied by Delmas-Beauvieux et al.^36^ remained unclear. The study^41^ lacked adequate identification and strategies for confounding factors but had clear inclusion criteria and valid outcome measurement. Notably, one study^42^ excelled in all aspects, including confounding factor identification and appropriate statistical analysis. Although some studies showed shortcomings in identifying and managing confounding factors, others displayed comprehensive methodological approaches. Among the case–control studies, one study^49^ featured comparable groups, appropriate matching, valid exposure measurement, and reliable outcomes, although the handling of confounding factors was unclear. Meanwhile, among the cohort studies, one study^35^ exhibited comparable groups, valid exposure measurement, and identification of confounding factors, but issues arose regarding incomplete follow-up and unexplored reasons for loss to follow-up. Similarly, in another study^43^, despite similar groups, valid exposure measurement, and identified confounding factors, uncertainties persisted regarding participant status, follow-up completeness, and strategies for addressing confounding factors.

### Qualitative synthesis

In studies involving nonpregnant individuals^14,32–39,41–43,45–47,49^, the levels of CAT were consistently lower in individuals infected with malaria compared with uninfected controls^14,32–34,37,38,41–43,49^. When examining severe malaria cases, CAT levels were lower in controls, but no significant difference was observed in CAT levels between severe and nonsevere malaria cases or between uncomplicated malaria and controls^46^. Furthermore, there was no significant difference in CAT levels between malaria cases and uninfected controls^35,36,39^. However, in contrast to these findings, some studies reported higher CAT levels in malaria-infected individuals compared with uninfected controls^45,47^. Additionally, no significant differences in CAT levels were observed between different parasite densities^36^ or between *P. falciparum* and *P. vivax* malaria^14^. Three studies compared the differences in CAT levels across various levels of severity of clinical malaria^38,43,46^. In all three studies, there was no observed difference in CAT levels between severe and nonsevere malaria cases.

Among the studies involving pregnant women^40,44,48^, Olushola et al. demonstrated lower CAT levels in malaria-infected individuals compared with uninfected controls during the first trimester. However, during the second and third trimesters, no significant difference in CAT levels was observed between malaria cases and uninfected controls^44^. Meanwhile, Tiyong Ifoue et al. reported lower CAT levels in malaria-infected individuals compared with uninfected controls during the third trimester, but no significant difference in CAT levels was found between malaria cases and uninfected controls during the first and second trimesters^48^. Moreover, Megnekou et al. reported no significant difference in CAT levels between malaria-infected individuals and uninfected controls at delivery^40^.

Among the studies that enrolled pregnant women and compared quantitative data on CAT levels between *Plasmodium*-infected and uninfected controls^40,44,48^, the number of available studies was limited. Consequently, no meta-analysis comparing CAT levels between *Plasmodium*-infected and uninfected controls could be conducted. In summary, among nonpregnant individuals, the available evidence suggests that CAT levels were generally lower in malaria-infected individuals compared with uninfected controls, although certain studies reported higher CAT levels in infected individuals. However, no significant differences in CAT levels were found between severe and nonsevere malaria or between uncomplicated malaria and controls. Regarding pregnant women, the levels of CAT exhibited variations across trimesters. Some studies reported lower CAT levels during the first trimester, whereas others indicated lower levels during the third trimester. At the time of delivery, no significant differences in CAT levels were observed between malaria-infected individuals and uninfected controls.

### Quantitative synthesis (meta-analysis)

**Figure 2:**
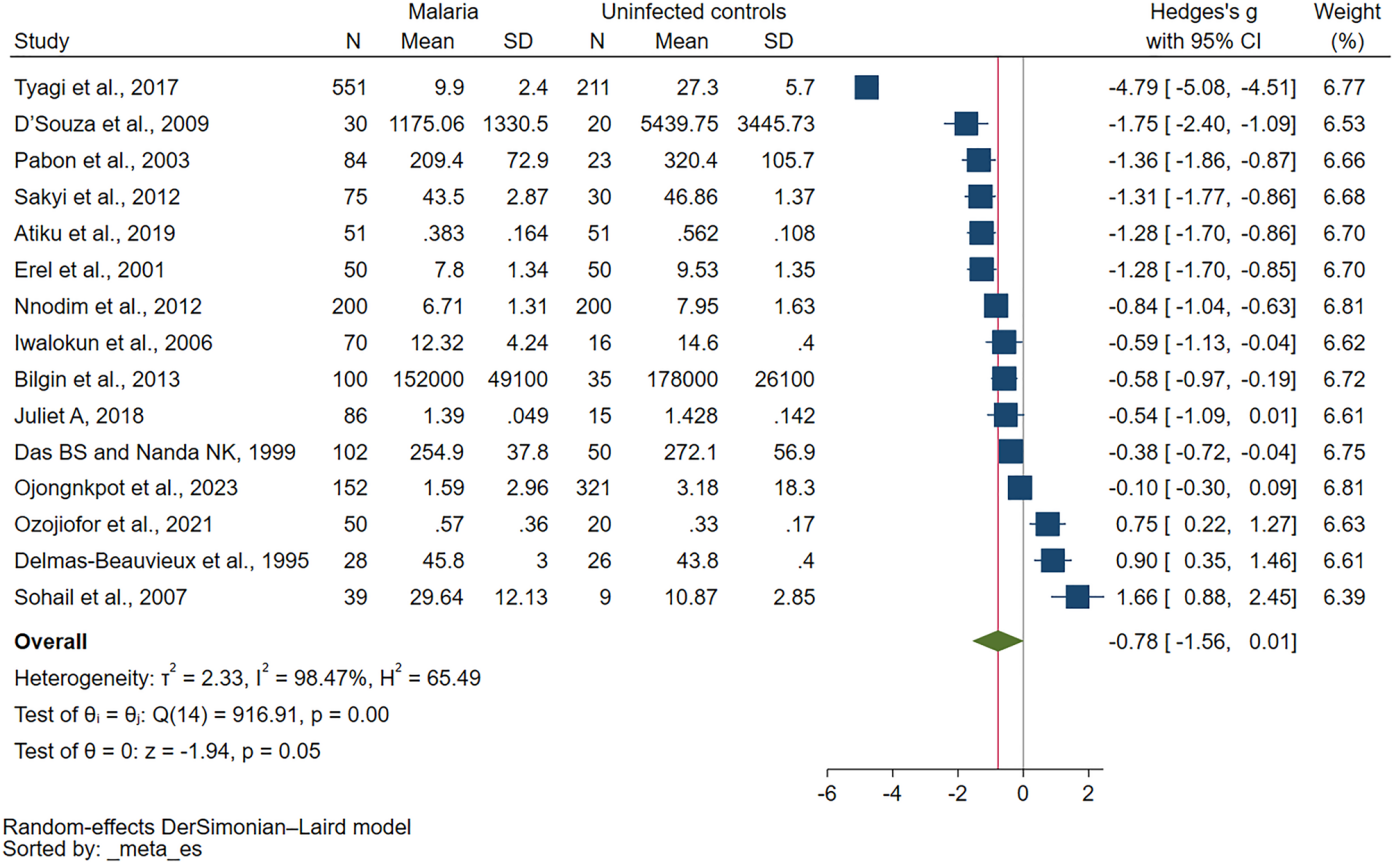
Forest plot shows the difference in CAT levels between patients with malaria and uninfected controls. The direction of the green diamond indicates CAT levels in relation to malaria infection: left of the middle line (0) suggests lower CAT levels in those with malaria compared with the uninfected controls, whereas right of the middle line suggests higher CAT levels in malaria-infected individuals. *CI* confidence interval, *N* number of participants, *SD* standard deviation.

Among the studies that enrolled nonpregnant individuals and reported quantitative data of CAT levels between malaria and uninfected controls^14,32–39,41,42,45–47,49^, the results of eight individual studies showed that CAT levels were lower in malaria-infected individuals compared with uninfected controls^32–34,37,38,41,46,49^. Meanwhile, no significant difference in CAT levels was observed between malaria and uninfected controls in four studies^14,35,39,42^. CAT levels were higher in malaria-infected individuals compared with uninfected controls in three studies^36,45,47^. Overall, the meta-analysis showed no difference in CAT levels between malaria and uninfected controls (*P* = 0.05, Hedges’ g: − 0.78, 95% CI: (− 1.56)–0.01, I^2^: 98.47, 15 studies, Fig. 2).

The findings from the meta-analysis indicated high heterogeneity, with an I^2^ value of 98.47% indicating significant variation in the results of the included studies. Meta-regression analysis and subgroup analyses were further performed. The meta-regression analysis, incorporating publication year, study design, continent, age group, *Plasmodium* species, clinical status, and diagnostic method for malaria revealed no significant influence of these covariates on the pooled effect estimate (*P* > 0.05, Table 2). This indicates that the heterogeneity observed in the effect estimates between studies could not be accounted for by these factors. Subgroup analyses examining publication year, study design, continent, age group, *Plasmodium* species, and diagnostic method for malaria demonstrated a notable finding. Among studies conducted in Africa, a significant difference in CAT levels was observed between malaria cases and uninfected controls (*P* = 0.02, Hedges’ g: − 0.57, 95% CI: − 1.02–(0.11), I^2^: 91.81, 7 studies, Table 3). Furthermore, studies that specifically focused on children exhibited significantly lower CAT levels in malaria cases compared with uninfected controls (*P* = 0.03, Hedges’ g: − 0.57, 95% CI: − 1.07–(− 0.07), I^2^: 87.52, 4 studies, Table3). Similarly, among studies utilizing microscopy alone as the method for diagnosing the presence of malaria parasites, a significant decrease in CAT levels was observed in malaria cases compared with those in uninfected controls (*P* = 0.04, Hedges’ g: − 0.91, 95% CI: − 1.76–(− 0.06), I^2^: 98.62, 13 studies, Table 3).

**Table 2:** Meta-regression results.

Meta-analysis of CAT	Covariates	*P* value	tau^2^	I^2^ (%)	R^2^ (%)	Number of studies
Patients with malaria vs. uninfected individuals	Publication years	0.70	2.60	98.7	0	15
	Study design	0.12	2.20	98.1	5.65	15
	Country	0.97	3.68	98.7	0	15
	Continent	0.55	2.12	98.4	9.10	15
	Age group	0.77	2.28	98.2	1.89	15
	*Plasmodium* species	0.61	2.58	98.7	0	15
	Clinical status	0.91	2.91	98.5	0	15
	Diagnostic method for malaria	0.39	2.34	98.5	0	15

**Table 3:** Subgroup analyses of CAT levels between malaria cases and uninfected controls.

Subgroup analyses	*P* value	Hedges’ g (95% CI)	I^2^	Number of studies
Publication year				
2010–2023	0.08	− 0.53 (− 1.78 to 0.72)	98.50	8
2000–2009	0.41	− 0.40 (− 1.36 to 0.55)	94.16	4
Before 2000	0.63	− 0.27 (− 1.34 to 0.81)	94.67	3
Study design				
Cross-sectional study	0.05	− 0.48 (− 0.95 to 0.00)	92.89	12
Case–control study	0.15	− 2.81 (− 6.69 to 1.06)	99.79	2
Cohort study	N/A	− 0.38 (− 0.72 to 0.04)	N/A	1
Continent				
Africa	0.02	− 0.57 (− 1.02 to (0.11))	91.81	7
Asia	0.21	− 1.20 (− 3.06 to 0.67)	99.16	6
Europe	N/A	0.90 (0.35 to 1.46)	N/A	1
South America	N/A	− 1.36 (− 1.86 to (− 0.87))	N/A	1
Age group				
Children	0.03	− 0.57 (− 1.07 to (− 0.07))	87.52	4
Adults	0.24	− 0.47 (− 1.27 to 0.32)	94.56	6
All age groups	0.38	− 1.55 (− 5.03 to 1.94)	99.58	3
Not specified	0.01	− 0.93 (− 1.66 to (− 0.20))	77.22	2
*Plasmodium* species				
* P. falciparum*	0.12	− 0.82 (− 1.87 to 0.22)	98.95	10
* P. vivax*	0.87	− 0.11 (− 1.42 to 1.20)	95.18	3
* P. falciparum*/*P. vivax*	< 0.01	− 1.50 (− 1.90 to (− 1.11))	0.00	2
Diagnostic method for malaria				
Microscopy	0.04	− 0.91 (− 1.76 to (− 0.06))	98.62	13
Microscopy/RDT	0.87	0.11 (− 1.15 to 1.36)	90.83	2

*CI* confidence interval, *N/A* not assessed, *RDT* rapid diagnostic test.

### Sensitivity analysis

**Figure 3:**
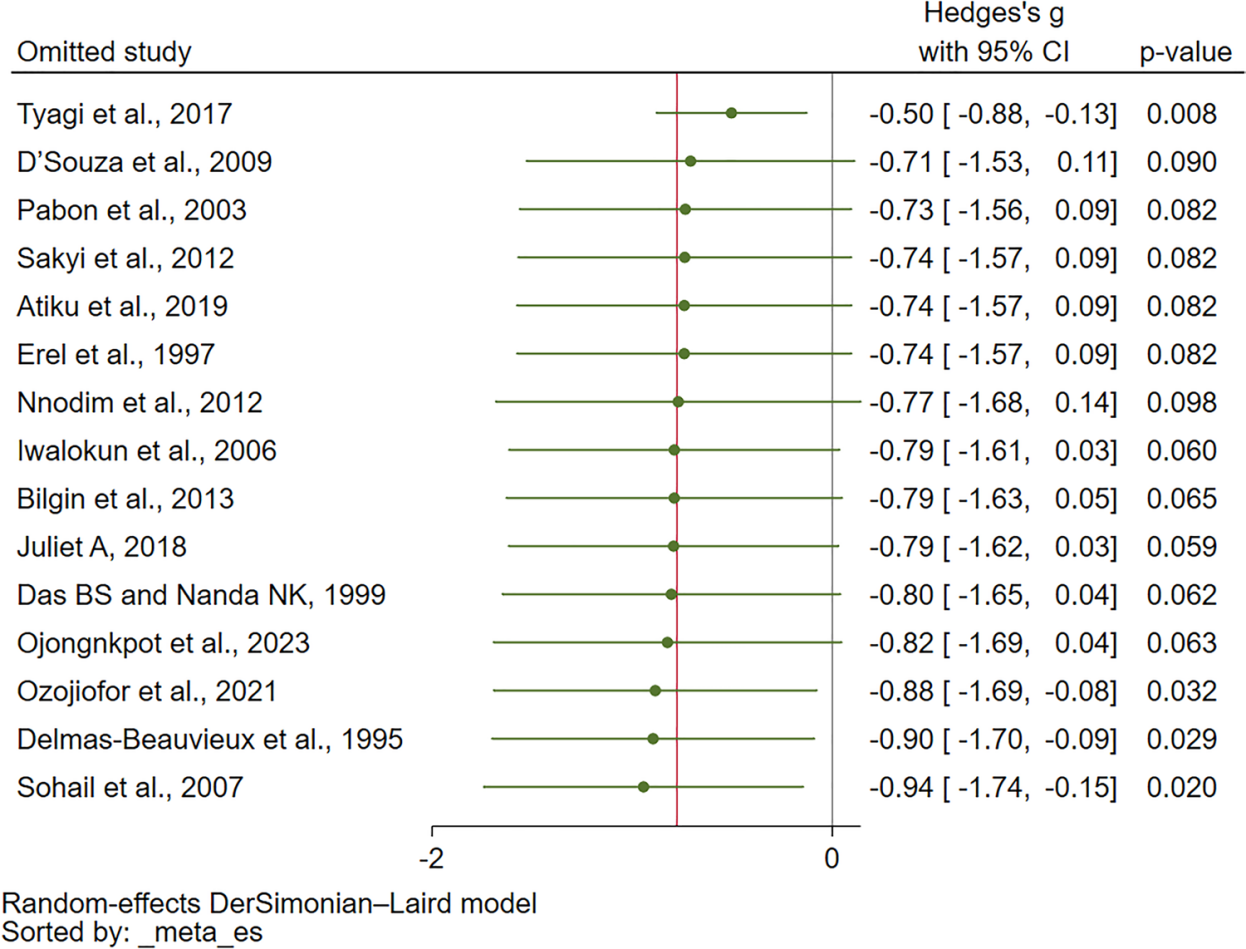
Leave-one-out method showing an outlier in the meta-analysis of the difference in CAT levels between patients with malaria and uninfected controls. The direction of the green dot indicates CAT levels in relation to malaria infection: left of the middle line (0) suggests lower CAT levels in those with malaria compared with the uninfected controls, whereas right of the middle line suggests higher CAT levels in malaria-infected individuals. *CI* confidence interval.

During the leave-one-out meta-analysis, it was identified that the studies conducted by Tyagi et al.^49^, Delmas-Beauvieux et al.^36^ Ozojiofor et al.^46^, and ^45^ Sohail et al.^47^ stood as outliers in the overall analysis. Upon excluding the results of these particular studies and rerunning the meta-analysis, a significant decrease in CAT levels among patients with malaria compared with those in uninfected controls was observed (*P* < 0.05, Fig. 3).

### Publication bias

**Figure 4:**
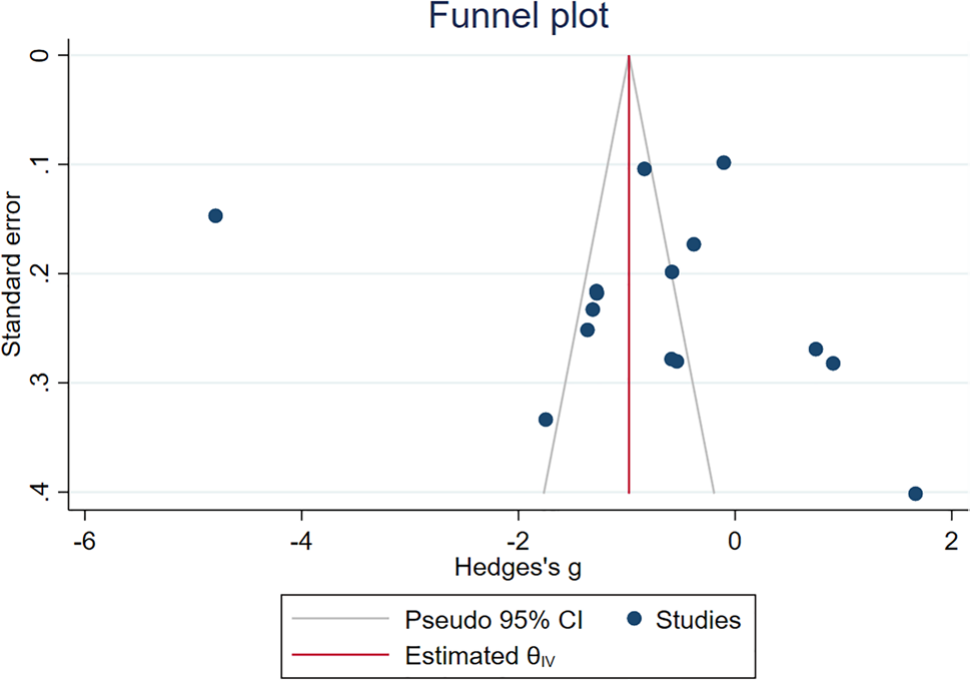
Funnel plot showing an asymmetrical distribution of the effect estimate of CAT levels between patients with malaria and uninfected controls. *CI* confidence interval.

The funnel plot exhibited asymmetry, and the results of Egger’s test indicated no significant presence of small study effects (*P* = 0.16, Fig. 4). However, employing the trim-and-fill method with imputation on the left side yielded a notable finding. It revealed a significant decrease in CAT levels among malaria cases compared with those in uninfected controls [Hedges’ g: − 0.98, 95% CI: − 1.07–(− 0.89)].

## Discussion

The present study investigated the levels of CAT in those infected with malaria compared with the uninfected controls. Among the studies that focused on nonpregnant individuals, a consistent finding was observed, with CAT levels being lower in individuals with malaria compared with uninfected controls^14,32–34,37,38,41–43,49^. This suggests that malaria infection may have an impact on CAT enzyme activity, leading to decreased levels. Interestingly, among the nonpregnant individuals, CAT levels were found to be lower in severe malaria cases compared with the controls. However, no significant difference in CAT levels was observed between severe malaria and nonsevere malaria or between uncomplicated malaria and controls^46^. Furthermore, three studies that compared the differences in CAT levels across various levels of clinical malaria^38,43,46^ demonstrated no observed difference in CAT levels between severe and nonsevere malaria cases. These findings suggest that the severity of the disease itself may not be the primary determinant of CAT levels in nonpregnant individuals with malaria. Furthermore, it is worth noting that some studies did not observe any significant difference in CAT levels between malaria-infected individuals and the uninfected controls^35,36,39^. In contrast, a couple of studies reported higher CAT levels in individuals with malaria compared with the uninfected controls^45,47^. These divergent findings may indicate variations in the study populations, methodology, or other factors that influence CAT levels in the context of malaria.

Subgroup analyses were performed to investigate specific factors that might contribute to the heterogeneity and divergent findings. Notably, among studies conducted in Africa, a significant difference in CAT levels was observed between malaria cases and uninfected controls. This finding suggests that geographical location may play a role in CAT levels among malaria-infected individuals. A possible explanation for the observed significant difference in CAT levels between malaria cases and uninfected controls in studies conducted in Africa could be the increased oxidative stress^10,50^ or impaired immune responses in children during the first years of life in Africa^51,52^, potentially reducing CAT levels. Additionally, the meta-analysis focusing specifically on children showed a significant decrease in CAT levels in malaria cases compared with that in the uninfected controls. This finding confirms that age group could be a factor influencing CAT levels. Similarly, the subgroup analysis that used microscopy alone as the method for diagnosing the presence of malaria parasites also showed a significant decrease in CAT levels in malaria cases compared with that in the uninfected controls. A possible explanation for the observed significant difference in CAT levels of the studies that used microscopy alone as the method for diagnosing the presence of malaria parasites is the fact that microscopy is a standard method for identifying such parasites. Thus, it highlights the importance of considering these factors when interpreting CAT levels in the context of malaria infection. Further research is needed to explore the underlying mechanisms and potential clinical implications of the observed differences in CAT levels among different populations and settings.

Among pregnant women, the studies demonstrated contrasting results. Olushola et al. showed lower CAT levels in pregnant women with malaria compared with the uninfected controls during the first trimester, but no significant difference in CAT levels was observed between malaria cases and uninfected controls during the second and third trimesters^44^. Conversely, Tiyong Ifoue et al. found lower CAT levels in pregnant women with malaria compared with the uninfected controls during the third trimester, whereas no significant difference was observed during the first and second trimesters^48^. These findings suggest that the impact of malaria on CAT levels during pregnancy may vary across trimesters. Additional research is needed to understand the underlying mechanisms and potential implications of these trimester-specific differences. Lastly, Megnekou et al. found no significant difference in CAT levels between pregnant women with malaria and the uninfected controls at the time of delivery^40^. This suggests that CAT levels may normalize or reach a comparable state between the two groups toward the end of pregnancy. Further investigations are necessary to elucidate the factors contributing to these observations and to explore the potential implications for maternal and fetal health.

Oxidative stress markers, such as malondialdehyde (MDA) have been reported to be positively correlated with malaria parasite density; meanwhile, negative correlations have been observed between parasite density and antioxidant enzymes, such as GSH, SOD, ascorbic acid, and CAT^42,53^. These results indicate that not only are CAT levels affected by *Plasmodium* infection but also other antioxidants tend to be lower. The association between catalase and other antioxidant systems in malaria is complex and interconnected. The activity of CAT can be influenced by the levels and functions of other antioxidant enzymes, and vice versa. Understanding the interplay between these systems is crucial for unraveling the mechanisms of oxidative stress and developing potential therapeutic interventions to mitigate the damage caused by malaria.

The present study had certain limitations. First, the results of the meta-analysis were highly heterogeneous and should be interpreted with caution. Second, the funnel plot in the meta-analysis suggested publication bias, so this should be taken into consideration when interpreting the results.

## Conclusion

This study provides valuable insights into the association between malaria infection and CAT enzyme levels, particularly in nonpregnant individuals. However, discrepancies in the results and the trimester-specific differences observed in pregnant women highlight the complexity of this relationship. Identifying and controlling for potential confounding factors can help in understanding the direct relationship between CAT levels and malaria. Understanding the variations in CAT levels in the context of malaria infection can offer insights to improve the diagnostic, therapeutic, and preventive strategies for malaria, particularly concerning the unique needs and vulnerabilities of pregnant women. Further, well-designed studies are essential to decode the intricacies of this relationship, which could have significant implications for understanding disease processes and improving patient care.

### Supplementary Information

Supplementary Table S1.

Supplementary Table S2.

Supplementary Table S3.

## Data Availability

All data relating to the present study are available in this manuscript and supplementary files.
