# Supplementary material for: Differences in catalase levels between malaria-infected individuals and uninfected controls: a systematic review and meta-analysis
Source: Sci Rep. 2023 Sep 5;13:14619. doi: 10.1038/s41598-023-41659-4 (PMC10480170; doi:10.1038/s41598-023-41659-4)
Supplement: Supplementary file 1 — Supplementary Table S1. [file 41598_2023_41659_MOESM1_ESM.docx]

**Differences in catalase levels between malaria-infected individuals and uninfected controls: A systematic review and meta-analysis**

Manas Kotepui^1^, Aongart Mahittikorn^2*^, Frederick Ramirez Masangkay^3^, Kwuntida Uthaisar Kotepui^1^*

^1^Medical Technology, School of Allied Health Sciences, Walailak University, Tha Sala, Nakhon Si Thammarat, Thailand

^2^Department of Protozoology, Faculty of Tropical Medicine, Mahidol University, Bangkok, Thailand

^3^Department of Medical Technology, Faculty of Pharmacy, University of Santo Tomas, Manila, Philippines

**^*^Corresponding author**

Manas Kotepui: manas.ko@wu.ac.th

Aongart Mahittikorn: [aongart.mah@mahidol.ac.th](mailto:aongart.mah@mahidol.ac.th)

Frederick Ramirez Masangkay: frederick_masangkay2002@yahoo.com

Kwuntida Uthaisar Kotepui: [kwuntida.ut@wu.ac.th](mailto:kwuntida.ut@wu.ac.th)

**Table S1. Search terms**

**General keywords**

catalase AND (malaria OR plasmodium OR “Plasmodium Infection“ OR “Remittent Fever“ OR “Marsh Fever“ OR Paludism)

PubMed 27 May 2023

| No. | Key concept | Search terms | Results |
| --- | --- | --- | --- |
| 1. | Catalase | (catalase[Text Word]) OR (catalase[MeSH Terms]) | 78,155 |
| 2. | Malaria | malaria[tw] OR plasmodium[tw] OR “Infections, Plasmodium“[Mesh] OR “Infection, Plasmodium“[Mesh] OR “Plasmodium Infection“[Mesh] OR “Remittent Fever“[Mesh] OR “Fever, Remittent“[Mesh] “Marsh Fever“[Mesh] OR “Fever, Marsh“[Mesh] OR Paludism[Mesh] | 117,984 |
| 3. | 1 AND 2 | (catalase[Text Word]) OR (catalase[MeSH Terms]) AND (malaria[tw] OR plasmodium[tw] OR “Infections, Plasmodium“[Mesh] OR “Infection, Plasmodium“[Mesh] OR “Plasmodium Infection“[Mesh] OR “Remittent Fever“[Mesh] OR “Fever, Remittent“[Mesh] “Marsh Fever“[Mesh] OR “Fever, Marsh“[Mesh] OR Paludism[Mesh]) | 191 |

Embase 27 May 2023

| No. | Key concept | Search terms | Results |
| --- | --- | --- | --- |
| 1. | Catalase | catalase:ti,ab,kw,de OR catalase/exp | 108,489 |
| 2. | Malaria | malaria:ti,ab,kw,de OR plasmodium:ti,ab,kw,de OR ‘Remittent Fever’:ti,ab,kw,de OR ‘Marsh Fever’:ti,ab,kw,de OR Paludism:ti,ab,kw,de OR malaria/exp | 155,929 |
| 3. | 1 AND 2 | (catalase:ti,ab,kw,de OR catalase /exp) AND (malaria:ti,ab,kw,de OR plasmodium:ti,ab,kw,de OR ‘Remittent Fever’:ti,ab,kw,de OR ‘Marsh Fever’:ti,ab,kw,de OR Paludism:ti,ab,kw,de OR malaria/exp) | 335 |

Scopus 27 May 2023

| No. | Key concept | Search terms | Results |
| --- | --- | --- | --- |
| 1. | Catalase | TITLE-ABS-KEY (catalase) | 130,846 |
| 2. | Malaria | TITLE-ABS-KEY ( malaria OR plasmodium OR "plasmodium infection" OR "remittent fever" OR "marsh fever" OR paludism ) | 156,580 |
| 3. | 1 AND 2 | ( TITLE-ABS-KEY (catalase) ) AND ( TITLE-ABS-KEY ( malaria OR plasmodium OR "plasmodium infection" OR "remittent fever" OR "marsh fever" OR paludism ) ) | 334 |

MEDLINE 27 May 2023

| No. | Key concept | Search terms | Results |
| --- | --- | --- | --- |
| 1. | Catalase AND Malaria | catalase AND (malaria OR plasmodium OR “Plasmodium Infection“ OR “Remittent Fever“ OR “Marsh Fever“ OR Paludism) | 197 |

Ovid 27 May 2023

| No. | Key concept | Search terms | Results |
| --- | --- | --- | --- |
| 1. | Catalase AND Malaria | catalase AND (malaria OR plasmodium OR “Plasmodium Infection“ OR “Remittent Fever“ OR “Marsh Fever“ OR Paludism) {No Related Terms} (ovid full text available and articles with abstracts and original articles) | 152 |

ProQuest 27 May 2023

| No. | Key concept | Search terms | Results |
| --- | --- | --- | --- |
| 1. | Catalase AND Malaria | catalase AND (malaria OR plasmodium OR “Plasmodium Infection“ OR “Remittent Fever“ OR “Marsh Fever“ OR Paludism) | 691 |
